# Maternal and neonatal outcomes in a treated versus non- treated cohort of women with Gestational Diabetes Mellitus according to the HAPO 5 and 4 criteria

**Published:** 2017-09

**Authors:** A-S Maryns, I Dehaene, G Page

**Affiliations:** Department of Obstetrics and gynecology, University Hospital of Ghent, 9000 Ghent, Belgium; Department of Obstetrics and gynecology, Jan Yperman Hospital, 8900 Yper, Belgium

**Keywords:** Gestational Diabetes Mellitus, screening, mildly glycemic aberrant pregnant women, HAPO 5 criteria, HAPO 4 criteria, IADPSG criteria

## Abstract

**Background:**

Our aim was to evaluate the treatment effect of gestational diabetes mellitus (GDM) according to the Hyperglycemia and Adverse Pregnancy Outcome group (HAPO) screening.

**Results:**

The prevalence of GDM, using HAPO 5 was 23.8%. Of these, 72.8% were treated. Comparison of outcomes between treated and untreated patients showed no differences. The prevalence of GDM according to HAPO 4 criteria was 16.9%. In the untreated group, there were more cases of (pre)eclampsia (P=0.038), more admissions to neonatal care department (P=0.036), pregnancy duration was shorter (P=0.05), and Apgar score at five minutes was significantly lower (P=0.019). The outcomes didn’t differ in the MAGG (midly aberrant glycemic group).

**Conclusions:**

Using HAPO 5 criteria in population-based screening doubled the prevalence of GDM. There were no differences between untreated and treated HAPO 5 and MAGG patients, while in the HAPO 4 group there might be a trend of therapy effectiveness.

## Introduction

Screening criteria for gestational diabetes mellitus (GDM) are a hot topic in perinatal care (Belhalima et al., 2015; Gupta et al., 2015; Sacks et al., 2015). The glycemic screening values have long been based upon cut-off values in a non-pregnant population (Carpenter and Coustan criteria) (Gupta et al., 2015; Sacks et al., 2015). In 2008, the Hyperglycemic And Pregnancy Outcome study (HAPO) confirmed a continuous relationship between obstetrical complications, perinatal morbidity, and blood glucose levels independent of BMI, maternal age, parity, and mean arterial pressure (Metzger et al., 2008; Coustan et al., 2010; Ethride et al., 2014; Gupta et al., 2015; Sacks et al., 2015; Billionet et al., 2017). As a consequence, the choice of cut-off values for the oral glucose tolerance test (OGTT) is inevitably arbitrary (Coustan et al., 2010; Gupta et al., 2015). The optimal screening method should compromise between over- and underestimation of the disease, and preferably be the most cost- effective strategy (Ohno et al., 2011; Gupta et al., 2015).

The International Association of Diabetes And Pregnancy Study Group (IADPSG) recommended, based on the results of the HAPO results, screening criteria for GDM based on the risk of pregnancy complications. The IADPSG consensus panel recommended the use of the HAPO 5 cut-off levels for a 75g glucose OGTT. These HAPO 5 cut-off values for the 75g glucose OGTT, taken in 2nd trimester between 24 and 28 weeks of amenorrhea, correspond with an odds ratio of 1.75 for pregnancy complications if left untreated. The HAPO 5 OGTT cut-off levels are 92 mg/dl at fasting time, 180mg/ dl after one hour and 153mg/dl after two hours. GDM is diagnosed in second trimester if at least one value equals or exceeds the cut-off values. The first trimester fasting cut-off value (92 mg/dl) was chosen by consensus by the IADPSG panel (Metzger et al., 2008).

The HAPO 4 criteria correspond with an odds ratio of two: 95 mg/dl at fasting, 191mg/dl at one hour interval, and 162mg/dl at two hour interval in the 2nd trimester OGTT. If one or more values exceed or equal the cut-off values, GDM is diagnosed. Using the HAPO 4 cut-off values, the prevalence of GDM would be lower and fewer patients would be treated. The benefit of treating patients with values between HAPO 4 and HAPO 5 values, the mildly aberrant glycemic values, is subject to discussion (Metzger et al., 2008; Belhalima et al., 2015; Gupta et al., 2015; Sacks et al., 2015).

Since publication of a ‘number needed to treat analysis’ that proved cost-effectiveness of HAPO 5 criteria, the World Health Organization (WHO), the International Federation of Gynecology and Obstetrics (FIGO) and the Canadian Diabetes Association promoted the HAPO 5 cut-off values in population-based screening (often shortly referred to as the IADPSG criteria) (Landon et al., 2009; Ohno et al., 2011; Gupta et al., 2015; Sacks et al., 2015). The WHO acknowledges that the evidence for adapting the IADPSG recommendations is low (Belhalima et al., 2015). In Flandres, the guidelines are not universally followed: a mixture of risk factor-based screening and different cut-off values are used.

Screening in a population-based manner instead of risk factor-based screening is debatable. Reported risk factors for GDM (and for overt diabetes) are a previous history of GDM, maternal age ≥ 40 years old, BMI > 35, history of type 2 diabetes in a first degree relative, a history of a large for gestational age baby (> 90th centile or birth weight > 4500grams), use of corticosteroids or antipsychotics, polycystic ovary syndrome, ethnicity like Mediterranean, South Asian, African Black, North African, Carribean, Middle Eastern, and Hispanic (Belhalima et al., 2015; Gupta et al., 2015). However, European Board & College of Obstetrics and Gynecology (EBCOG) stated that more data are needed to define the best predictive risk factors among European populations (Belhalima et al., 2015).

The National Institute of Health (NIH) and the American College of Obstetricians and Gynecologists (ACOG) favor a two-step screening protocol. Only if a glucose challenge test is positive (≥ 140 mg/dl) a 3-hourly OGTT test with 100g glucose is performed (with cut-off levels of 95 mg/ dl, 180 mg/dl, 155 mg/dl and 140 mg/dl) (Belhalima et al., 2015; Gupta et al., 2015).

The aim of our study was to evaluate the treatment effect (effectiveness) of GDM, in a population- based screening setting, according to the HAPO 5 and 4 screening criteria, as well as in patients with mildly aberrant glycemic values (MAGG).

## Methods

We conducted a prospective observational study. Data collection was performed from May 2012 until January 2015. This study was performed in the Jan Yperman Hospital in Ypres, Belgium, a peripheral center with 1200 deliveries a year. Informed consent was obtained for all patients for studying patient data in the electronic patient file of the hospital.

Screening was offered to every pregnant patient. Prenatal follow-up was not altered for the purpose of this study. Exclusion criteria were multiple pregnancies. At the first prenatal consultation, a fasting or random glycemia was measured. The risk factors for GDM were documented.

Diagnosis of GDM in the first trimester was defined as a fasting glycemia equal to or exceeding 92 mg/dl or a random glycemia equal to or exceeding 120 mg/dl. If fasting glycemia was equal to or more than 126 mg/dl, the diagnosis of pre-existing diabetes mellitus was made.

Between 24 and 28 weeks of amenorrhea a 75 gram OGTT was performed. Blood glucose levels were measured at fasting, after one, and two hours intervals. The HAPO 5 criteria were fulfilled in second trimester if one or more levels equaled or exceeded 92 mg/dl, 180mg/dl or 153mg/dl.

When diagnosed with GDM or pre-existing diabetes, the patient was referred to the endo- crinologist. HbA1c was measured in all patients. If the percentage equaled or exceeded 6.5%, the patient was categorized as having pre-existing diabetes mellitus.

Therapy in our center was performed using a step-up protocol. The first step was regular physical activity and diet. A diet with a maximum of 1800 kcal a day and a maximum of 40% carbohydrates was advised. Once a week, glucose levels were measured at seven different moments: fasting, pre-prandial, and 2 hours postprandial. The goal is fasting levels lower than 90 mg/dl, pre-prandial levels lower than 100 mg/dl and 2 hours postprandial levels lower than 120 mg/dl. The second step in therapy was insulin. Insulin was started if the first step failed or in the case of pre-existing diabetes.

Maternal and neonatal pregnancy outcome parameters were collected for each patient with GDM: weight gain, spontaneous labor, induction of labor, mode of delivery, pregnancy duration in days, pregnancy induced hypertension, (pre)eclampsia and HELPP syndrome, birth weight, Apgar scores at one and five minutes, large and small for gestational age babies (respectively above 90th percentile or lower than 10th percentile), shoulder dystocia, admission to neonatal care or to the neonatal intensive care department. Neonatal glycemia was only measured in the large for gestational age babies, and after use of insulin therapy. Because of the small number of events, a composite maternal outcome (occurrence of (pre)eclampsia, hypertension and HELLP syndrome, secondary and primary caesarean section, and assisted delivery) was preferred. A composite neonatal outcome was formulated in which large for gestational age babies, neonatal (intensive) care admissions, and the occurrence of shoulder dystocia were included.

These outcomes were compared between treated and untreated patients in the HAPO 5 group (population-based screening with HAPO 5 criteria), in the HAPO 4 group (population-based screening with HAPO 4 criteria) and in the group that had diabetes according to HAPO 5 but not according to HAPO 4. The latter was called the mildly aberrant glycemic group or MAGG.

In our study the following risk factors were considered to calculate the prevalence of GDM in case of risk factor-based screening: maternal age ≥ 35 years old, BMI higher than 30, history of diabetes in a first degree relative, a history of a large for gestational age baby, and a personal history of GDM.

Statistical analysis was performed with SPSS 24. The Fisher’s Exact Test was used for categorical variables and the independent sample t-test for continuous parameters. Additionally a binominal logistic regression was performed for the composite maternal outcome to adjust for confounders. A two sided p-value <0.05 was considered to indicate statistical significance at an α of 5% and β at 80%. Power analyses were performed post-analysis for the composite maternal outcome.

## Results

**Figure 1:**
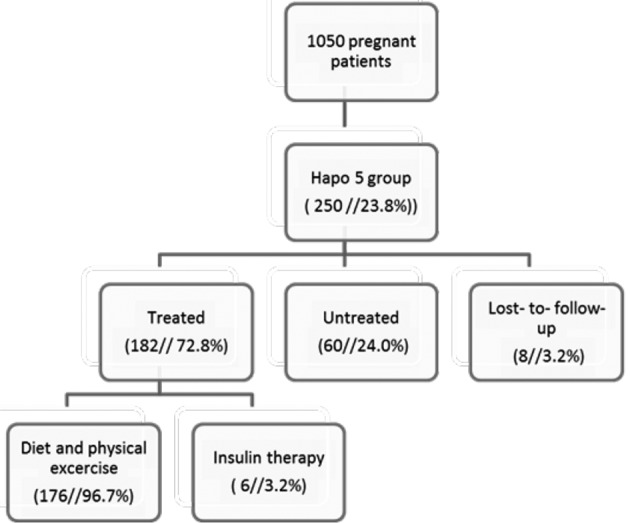
— summary of population-based screening with HaPO 5 criteria in an observational cohort

1050 pregnant women were included. The prevalence of GDM in the HAPO 5 group was 23.8% (250 women with eight patients lost-to-follow-up). The data of the lost-to-follow-up patients were not taken into account (two first trimester miscarriages, one second trimester miscarriage, two abortus arte provocatus, and three patients changing to another hospital). One hundred eighty two patients (72.8%) were treated by the endocrinology department with a diet and physical therapy. Insulin therapy was initiated in six women (3.2%). Due to various reasons, mainly patients unwilling to cooperate or gynecologists omitting referral, sixty women were untreated (24%) (Fig. 1).

Untreated patients had significantly lower glycaemia levels at one and two hours of the OGTT compared to the treated group (P = 0.014; P = 0.001). Untreated patients had a lower body mass index at pregnancy intake (P = 0.012), had a higher parity (P = 0.032), and were more often smokers (P = 0.047). Seventy-three patients (41.2%) were diagnosed with GDM based on first trimester fasting glycaemia. Maternal and neonatal outcomes between the two groups (HAPO 5 treated vs untreated) were compared and there were no statistically significant differences between the groups (Table I).

**Table I:** Outcomes in HAPO 5 group comparison treated vs untreated (ratio and prevalence in percentage, mean, P value treated versus untreated patients, mean difference and 95% confidence interval )

		treated	untreated	P-value	Mean difference (95% CI)
Maternal outcome	Induction of labor	40/182 (21.9%)	16/60 (26.6%)	0.512	
	Primary C-section	22/182 (12.1%)	5/60 (8.3%)	0.423	
	Vaginal birth	122/160 (76.2%)	44/55 (80.0%)	0.448	
	Assisted delivery	24/160 (15.0%)	6/55 (10.9%)	0.516	
	Secondary C-section	14/160 (8.8%)	5/55 (9.1%)	0.873	
	Pregnancy associated hypertension	14/182 (7.7%)	3/60 (5.0%)	0.479	
	(Pre)eclampsia	2/182 (10.9%)	2/60 (3.3%)	0.239	
	HELLP syndrome	2/182 (10.9%)	0/60 (0.0%)	0.415	
	Composite maternal outcome	94/182 (51.6%)	32/60 (53.3%)	0.821	
Neonatal outcome	Birth weight (grams)	3335.35	3231.10	0.200	81.17 (-264.15 - 55.65)
	Pregnancy duration (days)	273.98	272.32	0.153	-1.67 (5.40 - 2.07)
	APGAR score 5 min	9.49	9.31	0.088	-0.11 (-0.41- 0.03)
	Large for gestational age (LGA)	21/182 (11.5%)	4/60 (6.7%)	0.282	
	Neonatal care department	18/182 (9.9%)	0/60 (0.0%)	0.083	
	Neonatal intensive care department	2/182 (1.1%)	3/60 (5.0%)	0.065	
	Shoulder dystocia	3/182 (1.6%)	0/60 (0.0%)	1.000	
	Composite neonatal outcome	50/182 (27.5%)	17/60 (28.3%)	1.000	

The composite maternal outcome in the untreated group was 31.7% (19 out of 60 patients) (control event rate), and 38.5% (70 out of 182) in treated patients (experimental event rate). This could suggest overtreatment but the difference is not statistical significant (P = 0.360). We considered a 25% reduction in composite maternal outcome as clinically beneficial. Post hoc power analysis revealed that at least 1308 patients were needed in a 3/1 enrollment ratio; in the study there were 182 treated patients versus 60 untreated patient. A logistic regression was performed for the composite maternal outcome which adjusted for BMI, smoking, parity and nulliparity. The odds ratio of the composite maternal outcome depending on treatment was 0.652 (CI 0.342 – 1.241) (P = 0.193) (Table II).

**Table II:** Multivariate logistic regression for composite maternal outcome in HAPO 5, HAPO 4 and MAGG

HAPO 5	Odds ratio (95% CI)	P-value
Body mass index	1.034 (0.982 - 1.088)	0.203
Nulliparity ( yes or no )	1.695 (0.742 - 3.875)	0.211
Parity	0.772 (0.503 - 1.185)	0.237
Smoking ( yes or no )	0.663 ( 0.310 -1.419)	0.290
Treatment (yes or no)	0.652 (0.342-1.241)	0.193
HAPO 4	Odds ratio (95% CI)	P-value
Body mass index	1,008 (0.946 – 1.074)	0.805
Nulliparity ( yes or no )	1,509 (0.500 – 4.557)	0.465
Parity	0.665 (0.357 – 1.241)	0.200
Smoking ( yes or no )	1.194 (0.462 – 3.084)	0.715
Treatment (yes or no)	0.728 (0.303 – 1.753)	0.480
MAGG	Odds ratio (95% CI)	P-value
Body mass index	1.005 (0.909 – 1.112)	0.921
Nulliparity ( yes or no )	7.107 (0.716 – 70.510)	0.094
Parity	1.154 (0.280 – 4.750)	0.843
Smoking ( yes or no )	1.112 (0.280 – 4.425)	0.880
Treatment (yes or no)	1.949 (0.601 – 6.320)	0.266

The incidence of the composite neonatal outcome in the untreated group was 15/60 (25%) and in the treated group 40/182 (22%). The difference in our cohort was not statistical significant (P = 0.723). Risk factor-based screening using the HAPO 5 criteria resulted in a 10% prevalence of GDM (103 women).

**Figure 2:**
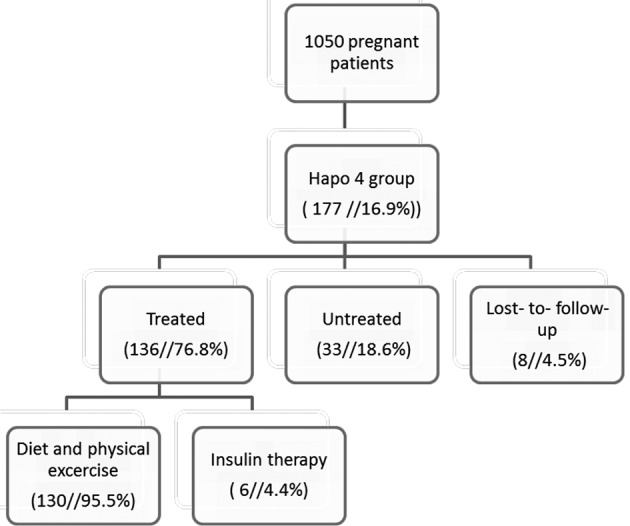
— Summary of population-based screening method with HAPO 4 criteria in an observational cohort

In the HAPO 4 group, the prevalence of GDM was 16.9% (177 women with eight patients lost-to-follow-up). One hundred and thirty six cases (76.8%) were treated for GDM and six patients received insulin therapy (4.4%). Thirty-three patients (18.6%) were not treated (Fig. 2). In the HAPO 4 group, the same 73 patients were diagnosed with GDM based on first trimester fasting glycaemia equaling or exceeding 92 mg/dl (41.2%). The untreated subgroup had significantly lower glycaemia levels at one and two hours of the OGTT than the group that received treatment (P = 0.014; P = 0.007). Untreated patients had a lower body mass index at pregnancy intake (P = 0.092), had a higher parity (P = 0.038), and smoked more (P = 0.268).

The composite maternal outcome in the untreated group was 36.4% (12 out of 33) and in the treated group 36% (49 out of 136). The difference in our cohort was not statistical significant (P= 1.000). (Table III) We considered a 25% reduction in composite maternal outcome to be clinically relevant. Post hoc power analysis revealed that at least 1347 patients were needed in a 4.5/1 enrollment ratio; in the study there were 136 treated patients versus 33 untreated patients. Logistic regression suggested an OR of treatment of 0.728 (0.303- 1.753) (P = 0.480) if adjusted for BMI, smoking, parity in general, and nulliparity or multiparity (Table II)

**Table III:** Outcomes in HAPO 4 group comparison treated vs untreated (ratio and prevalence in percentage, mean, P value treated versus untreated patients, mean difference and 95% confidence interval )

		treated	untreated	P-value	Mean difference (95% CI)
Maternal outcome	Induction of labor	31/136 (22.7%)	11/33 (33.3%)	0.209	
	Primary C-section	18/136 (13.2%)	3/33 (9.1%)	0.517	
	Vaginal birth	93/118 (78.8%)	24/30 (80.0%)	0.685	
	Assisted delivery	16/118 (13.6%)	3/30 (10.0%)	0.663	
	Secondary C-section	9/118 (7.6%)	3/30 (10.0%)	0.620	
	Pregnancy associated hypertension	11/136 (8.1%)	3/33 (9.1%)	0.851	
	(Pre)eclampsia	1/136 (0.73%)	2/33 (6.1%)	**0.038**	
	HELLP syndrome	0/136 (0.0%)	0/ 33 (0.0%)	0.491	
	Composite maternal outcome	69/136 (50.7%)	20/33 (60.6%)	0.308	
Neonatal outcome	Birth weight (grams)	3357.94	3228.28	0.202	-129.66 (-329.56 - 70.24)
	Pregnancy duration (days)	274.44	271.85	**0.05**	-2.60 (-7.62 - 2.42)
	APGAR score 5 min	9.48	9.13	**0.019**	-0.35 (-0.65 - -0.037)
	Large for gestational age (LGA)	15/136 (11.0%)	3/33 (9.1%)	0.746	
	Neonatal care department	9/136 (6.7%)	6/33 (18.2%)	**0.036**	
	Neonatal intensive care department	2/136 (14.7%)	2/33 (6.1%)	0.120	
	Shoulder dystocia	1/136 (0.73%)	0/33 (0.0%)	1.0	
	Composite neonatal outcome	33/136 (24.2%)	10/33 (30.3%)	0.507	

The incidence of the composite neonatal outcome in the untreated group was 9/33 (27.3%) and in the treated group 25/136 (18.4%) (P = 0.332). In the untreated group, there were more cases of (pre) eclampsia (P = 0.038), pregnancy duration was shorter (P = 0.05), more neonates were admitted to neonatal care (P = 0.036), and Apgar score at five minutes was significantly lower (P = 0.019) (Table III). There might be a clear trend of therapy effectiveness in the treated group. The pregnancy duration in the HAPO 4 treated group was 39 weeks and four days and was the longest of all subgroups. There weren’t more inductions of labor (P = 0.742). If a risk factor-based screening would have been performed with HAPO 4 values, the prevalence of GDM would have been 7.9% (83 patients).

The group with GDM according to HAPO 5 but not to HAPO 4 criteria in a population-based screening method was called the mildly aberrant glycemic group (MAGG) and consisted of 73 patients. Forty six (63%) were treated for GDM. Untreated patients did not significantly differ for BMI, smoking or parity with p-values of 0.572, 0.16 and 0.466 respectively. The glycemic values nor the outcomes differed significantly between the treated and untreated group.

The composite maternal outcome in the untreated group was 7/27 (25.9%) and in the treated group 21/46 (45.7%). The prevalence of the composite maternal outcome was almost doubled following treatment but the difference was not statistical significant (P = 0.135). We considered a 25% reduction in composite maternal outcome to be clinically relevant. Post hoc power analysis revealed that at least 1452 patients were needed in a 2/1 ratio; there were 46 treated patients versus 27 untreated patients. (Table IV). Additionally the logistic regression for the composite maternal outcome which adjusted for BMI, smoking, parity, and nulliparity or multiparity was not significant for treatment (P= 0.266). The calculated odds ratio was 1.949 (CI 0.601–6.320) (Table II).

**Table IV:** Outcomes in MAGG comparison treated vs untreated (ratio and prevalence in percentage, mean, P value treated versus untreated patients, mean difference and 95% confidence interval )

		treated	untreated	P-value	Mean difference (95% CI)
Maternal outcome	Induction of labor	10/46 (21.7%)	5/27 (18.5%)	0.742	
	Primary C-section	4/46 (8.7%)	2/27 (7.4%)	0.847	
	Vaginal birth	29/42 (69.0%)	20/25 (80.0%)	0.432	
	Assisted delivery	8/42 (19.0%)	3/25 (12.0%)	0.469	
	Secondary C-section	5/42 (11.9%)	2/25 (8.0%)	0.628	
	Pregnancy associated hypertension	3/46 (6.5%)	0/27 (0.0%)	0.175	
	(Pre)eclampsia	1/46 (2.2%)	0/27 (0.0%)	0.440	
	HELLP syndrome	2/46 (4.3%)	0/33 (0.0%)	0.272	
	Composite maternal outcome	25/46 (54.3%)	12/27 (44.4%)	0.472	
Neonatal outcome	Birth weight (grams)	3268.57	3234.44	0.560	-34.12 (-325.53 - 257.28)
	Pregnancy duration (days)	272.63	272.89	0.354	0.26 (-5.47 - 5.99)
	APGAR score 5 min	9.52	9.54	0.865	-0.03 (-0.36 - 0.31)
	Large for gestational age (LGA)	6/46 (13.0%)	1/27 (3.7%)	0.191	
	Neonatal care department	9/46 (19.5%)	5/27 (18.5%)	0.913	
	Neonatal intensive care department	0/46 (0.0%)	1/27 (3.7%)	0.189	
	Shoulder dystocia	2/46 (4.3%)	0/27 (0.0%)	0.527	
	Composite neonatal outcome	18/46 (39.1%)	7/27 (25.9%)	0.312	

The incidence of the composite neonatal outcome in the untreated group was 6/27 (22.2%) and in the treated group 15/46 (32.6%) (P = 0.427). We noted one patient with pregnancy induced hypertension, one with (pre)eclampsia, two patients with HELPP syndrome, two cases of shoulder dystocia and one concomitant clavicle fracture in the treated group (Table IV).

## Discussion

The IADPSG recommends a population-based screening for GDM with fasting or random glycaemia control in first trimester and an OGTT in second trimester. The detection of pre-existing diabetes in the first trimester was recommended due to a strong correlation between pre-existing diabetes and adverse pregnancy outcomes (Belhalima et al., 2015; Billionet et al., 2017). As threshold for GDM diagnosis in first trimester the IADPSG proposed ≥92mg/dl, following the HAPO study the panel promoted using HAPO category cut-off values for second trimester which corresponds with an OR of 1.75 (values of ≥ 92 mg/dl, ≥ 180mg/dl or ≥ 153mg/ dl) (Gupta et al., 2015). The IADPSG and HAPO study didn’t take long term maternal and neonatal risks into account in ascertaining these cut-off values (Coustan et al., 2010).

Our results show that population-based screening according to the HAPO 5 criteria in first and second trimester doubled the prevalence of GDM in comparison with a risk factor-based screening. In Flandres, there is the VDV-VVOG-Domus Medica consensus 2012 but the method used mainly depends on the hospital protocol (Benhalima, 2012). In France, the IADPSG criteria were accepted on a risk factor-base, while in the Netherlands screening is risk factor-based but with other OGTT cut-off values (Billionet et al., 2017). Compared to other papers in which a population-based screening was performed our prevalence of GDM is even higher: Sacks et al, (2015) noted a GDM prevalence of 19.2%, the IADPSG panel estimated a prevalence of 17.8% versus 23.8% in our study (11.4% versus 16.9% if using HAPO 4 criteria ) (Metzger et al., 2010; Sacks et al., 2015; Billionet et al., 2017).

Further prospective trials in European populations are needed to show which screening therapy is most cost-effective: risk factor-based, population-based screening, two-step screening and which OGTT cut-off values have to be applied (Belhalima et al., 2015).

The concern that a population-based screening with HAPO 5 criteria would lead to over treatment by provoking more inductions, more caesarean sections, and a higher number of neonatal admissions could not be confirmed in our study (Landon et al., 2009).

Landon published an RCT performed in the USA in which treated and untreated mild gestational diabetic women (positive GCT test but fasting glycemia beneath 95mg/dl and two or more exceeding levels of a 100g OGTT) were compared: a reduction of fetal macrosomia, shoulder dystocia, cesarean delivery and hypertensive disorders was seen following treatment (Landon et al., 2009). The ACHOIS study (NNT = 34 to prevent any serious perinatal complication and NNH = 11 for induction of labor or admission to neonatal care) equally emphasized that there was a modest benefit of treatment of mild gestational diabetes. In this study, mild gestational diabetes was defined as having one or more risk factors for GDM or a positive GCT and 75 g OGTT in which fasting glycemia was beneath 140mg/dl and at 2 hours between 140 mg/dl and 200mg/dl (Crowther et al., 2015). So, the IADPSG criteria were not applied in these studies and new studies should be performed to detect the clinical benefit of treating mild gestational diabetes.

The National Institute for Health and Excellence care (NICE) of the United Kingdom adapted their guidelines in 2015 from a two-step approach to the use of a one-step 75 g OGTT in a risk factor-based screening. The cut-off levels were a fasting plasma glucose level of 100mg/dl and a 2-hour plasma glucose level of 140mg/dl (NICE Guidelines, 2015; Billionet et al., 2017;). The ACOG still recommends a two-step screening with a glucose challenging test (Gupta et al., 2015).

A cost-effectiveness analysis of Ohno et al. revealed that treatment of GDM following population- based screening with HAPO 5 values was cost- effective if the incremental cost of treatment was below 1330$ (Ohno et al., 2011). It is uncertain if this economic conclusion can be extrapolated to the Flemish population. Our study didn’t reveal any significant benefit of therapy in the HAPO 5 group nor in the MAGG. There might be a trend of therapy bene t by screening with HAPO 4 values in a population-based screening because of statistic significant differences in secondary outcome parameters. In the retrospective cohort study by Sacks et al. (2015) the risks of (pre)eclampsia, preterm delivery, primary cesarean delivery, shoulder dystocia, higher birth weight, large for gestational age, and neonatal hypoglycemia were significantly higher in the HAPO 4 group after adjustment for confounders. The MAGG had a significantly greater risk of higher birth weight and large for gestational age in comparison with women without GDM (Sacks et al., 2015). Their data demonstrated more adverse outcomes in subgroups with higher glucose levels, reflecting again the continuous relationship between complications and glycemia levels (Sacks et al., 2015).

There are critical remarks on our study: a prospective cohort study is a rather weak study design, prone to many confounding variables and there was no randomisation process. Power analyses were only performed after analysis and revealed insufficient inclusions (60 patients didn’t receive therapy for various reasons and there was no intention of not treating GDM patients), and the number of studied events were low.

Secondly, optimal treatment of the gestational diabetes patient should lead to a healthy normo- glycemic newborn and prevent complications. However, glycemic values of newborns at time of delivery and/or within 2 hours following delivery were not obtained in all cases and for that reason not analysed.

Thirdly, the indication for induction of delivery was not always specified, neither were the reasons for neonatal admissions, or primary Caesarean section. Induction of delivery or primary Caesarean section for an estimated large for gestational age baby could result in a newborn with greater risk of a postnatal hypoglycemia in case of prematurity.

Fourthly, in several cases the OGTT was performed before 24 weeks or after 28 weeks of gestation, possibly causing over- or under estimation; Fiftly, the number of patients treated with insulin was low (3.2%) in comparison with other publications (Billionet et al., 2017). Sixthly there is a possible intervention bias : the effect of intervention may not reduce clinical endpoints such as pre-eclampsia or hypoglycemia but on the long-term the benefit for the mother-child dyad of supplementary medical attention (a healthy diet and lifestyle changes) is not known. Finally the outcomes of the patients without GDM were not analysed.

There is a trend of screening for pregnancy related diseases in the first trimester of pregnancy, allowing to start treatment as early as possible and hence, yielding better outcomes. The recommendation of the IADPSG that first trimester fasting glucose levels equaling or exceeding 92mg/dl is classified as GDM is based on consensus instead of evidence. The NNT and NNH if fasting first trimester cut- off would have been ≥95 mg/dl (HAPO 4) has not yet been studied. Nanda et al. (2011) proposed a universal OGTT in first trimester with other cut- off values. Because pregnancy is a diabetogenic condition and glycemia levels are related to gestational age, the cut-off values should have to be lower in the first trimester. Alternatively, screening in second trimester could also be done before 24 weeks.

## Conclusions

Introducing a population-based screening with HAPO 5 criteria in a Flemish hospital doubles the prevalence of GDM. However, there were no statistical significant differences between treated and untreated HAPO 5 patients. If screening would have been performed with HAPO 4 criteria there seemed to be a trend of therapy effectiveness. There were no statistical differences between treated and untreated patients in the MAGG group. Study design and low numbers of obstetrical and neonatal complications hamper making firm conclusions about treatment effectiveness. Further prospective randomized research to the optimal cut-off values for screening and treating GDM is needed.

## Conflict of interest

There are no conflicts of interest.
